# Comparative toxicological assessment of 2 bisphenols using a systems approach: evaluation of the behavioral and transcriptomic responses of *Danio rerio* to bisphenol A and tetrabromobisphenol A

**DOI:** 10.1093/toxsci/kfae063

**Published:** 2024-05-10

**Authors:** Michael G Morash, Morgan W Kirzinger, John C Achenbach, Ananda B Venkatachalam, Jessica Nixon, Susanne Penny, Joëlle Pinsonnault Cooper, Deborah E Ratzlaff, Cindy L A Woodland, Lee D Ellis

**Affiliations:** Aquatic and Crop Resource Development, National Research Council of Canada, Halifax, NS B3H 3Z1, Canada; Aquatic and Crop Resource Development, National Research Council of Canada, Saskatoon, SK S7N 0W9, Canada; Aquatic and Crop Resource Development, National Research Council of Canada, Halifax, NS B3H 3Z1, Canada; Aquatic and Crop Resource Development, National Research Council of Canada, Halifax, NS B3H 3Z1, Canada; Aquatic and Crop Resource Development, National Research Council of Canada, Halifax, NS B3H 3Z1, Canada; Human Health and Therapeutics, National Research Council of Canada, Halifax, NS B3H 3Z1, Canada; New Substances Assessment and Control Bureau, Health Canada, Ottawa, ON K1A 0K9, Canada; New Substances Assessment and Control Bureau, Health Canada, Ottawa, ON K1A 0K9, Canada; New Substances Assessment and Control Bureau, Health Canada, Ottawa, ON K1A 0K9, Canada; Aquatic and Crop Resource Development, National Research Council of Canada, Halifax, NS B3H 3Z1, Canada

**Keywords:** endocrine disruption, transcriptomics, RNA-seq, zebrafish

## Abstract

The zebrafish (*Danio rerio*) is becoming a critical component of new approach methods (NAMs) in chemical risk assessment. As a whole organism *in vitro* NAM, the zebrafish model offers significant advantages over individual cell-line testing, including toxicokinetic and toxicodynamic competencies. A transcriptomic approach not only allows for insight into mechanism of action for both apical endpoints and unobservable adverse outcomes, but also changes in gene expression induced by lower, environmentally relevant concentrations. In this study, we used a larval zebrafish model to assess the behavioral and transcriptomic alterations caused by subphenotypic concentrations of 2 chemicals with the same structural backbone, the endocrine-disrupting chemicals bisphenol A and tetrabromobisphenol A. Following assessment of behavioral toxicity, we used a transcriptomic approach to identify molecular pathways associated with previously described phenotypes. We also determined the transcriptomic point of departure for each chemical by modeling gene expression changes as continuous systems which allows for the identification of a single concentration at which toxic effects can be predicted. This can then be investigated with confirmatory cell-based testing in an integrated approach to testing and assessment to determine risk to human health and the environment with greater confidence. This paper demonstrates the impact of using a multi-faceted approach for evaluating the physiological and neurotoxic effects of exposure to structurally related chemicals. By comparing phenotypic effects with transcriptomic outcomes, we were able to differentiate, characterize, and rank the toxicities of related bisphenols, which demonstrates methodological advantages unique to the larval zebrafish NAM.

The development of new approach methods (NAMs) for chemical risk assessment is critical as governments move away from animal testing. NAMs take advantage of a number of models including cell-based, computational (*in silico*), adverse outcome pathways, and other nonanimal replacement strategies (Bajard et al. 2023). Although information gained from cell-line testing can provide valuable insight for toxicological mechanisms in individual cell types, testing on multiple cell lines fails to provide a system-level approach for chemical hazard assessment. As such, cell-based assays/*in silico* models for use in risk assessment may be unable to capture apical endpoints/adverse outcomes (Brescia et al. 2023). An important advantage of the whole organism approach, with completely formed and interconnected organ systems, is that it allows for the assessment of each of the 4 main components of chemical disposition (ADME), and not just metabolism, as the chemical traverses the entire whole organism prior to elimination. This allows for evaluation of toxicokinetic effects which (i) cannot be assessed using standard NAMs, such as cell-based lines as they may lack metabolic competence (Magurany et al. 2023; Parish et al. 2020); (ii) incorporate effects on signaling molecules (i.e., kinases) and on transporter proteins (i.e., multi-drug-resistant (mdr) proteins) involved in all ADME steps; and (iii) predict effects of chemical bioaccumulation in adipose tissue which can potentiate toxicity in fasting conditions (Casarett et al. 2001).

Accurate evaluation of chemical effects in order to establish an adverse outcome must include the above parameters, which at this time is only possible using a systems approach (whole-organism) model. Zebrafish embryo/larvae models have been developed and accepted as a NAM for environmental toxicological testing (OECD 2013) and given that in Canada and the EU it is considered an *in vitro* (nonanimal) model up to 5 days post fertilization, they offer a unique series of benefits (Batt et al. 2005; Directive 2010). They provide a system-level approach to toxicological testing that has been used to allow both toxicokinetic and transcriptomic paradigms to successfully predict chemical toxicity (Lai et al. 2021; Gou et al. 2023; Morash et al. 2023). In this paper we report on toxicity testing conducted on 2 related chemicals, bisphenol A (BPA) and tetrabromobisphenol A (TBBPA), using the well-described general and behavioral toxicity (GBT) larval exposure paradigm (Achenbach et al. 2020; Morash et al. 2023), which assays toxicity once organogenesis and organ patterning has been established. Compared with the OECD 236 fish embryo toxicity assay, the exposure window in the GBT assay (72- to 120-h postfertilization) prevents issues such as hatching defects, which could have downstream nonchemical mediated effects on behavior and development, along with perturbations in early development processes that may complicate assessment of phenotype causes.

BPA is a demonstrated endocrine-disrupting chemical (Almeida et al. 2018), which can interact with androgenic and thyroid receptors (Rochester 2013). In zebrafish, BPA induces changes in a number of estrogen-related pathways (Kubota et al. 2023) and in multiple aspects of the thyroid hormone system (Lee et al. 2019). Phenotypically, BPA toxicity manifests in several well-described pathologies, including imbalances in lipid utilization that lead to yolk resorption defects (Martinez et al. 2020) and perturbations in metabolic pathways (Qiu et al. 2019). Similarly, issues of pericardial edema (Achenbach et al. 2020) and decreased heart rate (Sundarraj et al. 2021) are well-documented. Mechanistically, BPA exposure has been shown to induce production of reactive oxygen species (ROS), leading to alterations in immune function (Qian et al. 2023), changes in brain morphology (Yang et al. 2023), and the expression of a number of developmental genes (Heredia-García et al. 2022). Although BPA-induced locomotor alterations have been described (Wang et al. 2021; Gu et al. 2022), there is not yet a consensus on the precise source and extent of these deficits (Coumailleau et al. 2020; Scaramella et al. 2022).

TBBPA is a brominated derivative of BPA, used mainly as a flame retardant in electronic plastics and resins (Malkoske et al. 2016). Exposure to TBBPA has been documented to occur via water contamination, in TBBPA-containing dust, and through breastmilk (Feiteiro et al. 2021). Although there is an extensive documentation of the results of BPA exposure on zebrafish, less is known regarding TBBPA. The endocrine-disrupting activity of TBBPA is mediated primarily through interactions with the thyroid system, including changes in T3/T4 ratio, and potentially expression of thyroid-related genes (Chen et al. 2016; Zhu et al. 2018). A limited number of phenotypes observed in zebrafish have been ascribed to TBBPA exposure, including a reduction in eye size (Baumann et al. 2016), hematopoietic toxicity (Pang et al. 2020), and hatching (Achenbach et al. 2020). Although deficits in coiling have been noted (Zhu et al. 2022), there is contradictory evidence for locomotor effects (Kacew and Hayes 2020).

In this paper, we demonstrate several advantages of our larval zebrafish platform by differentiating toxicological mechanisms of 2 highly similar chemicals, BPA and TBBPA. In addition to expanding upon previously reported differences in behavioral effects, using a whole transcriptome approach, we identify both common and distinct pathways associated with the well-documented phenotypic profiles. Lastly, we used a transcriptomic benchmark dose (BMD) approach to determine point of departure (POD) limits and identify the most sensitive pathways. This work highlights the importance of incorporating transcriptomics into the larval zebrafish model as it becomes a sensitive tool for chemical risk assessment.

## Materials and methods

### Chemicals

BPA (CAS No. 80-05-7, purity>99%), TBBPA (CAS No. 79-94-7, purity ≥99%), and DMSO (Cat No. D8418, purity ≥99%) were all purchased from Sigma-Aldrich (Oakville, ON, Canada). Both bisphenols were dissolved in DMSO at a stock concentration of 200 mM. All stocks were stored at −20°C.

### Animal husbandry

The afternoon prior to breeding, multiple male and female zebrafish (AB/Tub hybrids, previously reported; Morash et al. 2023) from different parental lineages were placed in 2–4 recirculating tanks containing dividers to separate the sexes. The next morning, dividers were removed and breeding allowed to occur for 1 h before the egg clutch was collected. For all experiments, experimental replicates used different clutches. Zebrafish embryos were kept in E3 media (5 mM NaCl, 0.17 mM KCl, 0.33 mM CaCl_2_–2H_2_O, 0.33 mM MgSO_4_–7H_2_O) in 10 mm × 150 mm disposable polystyrene petri dishes at 28.5°C and sorted for fertilization at 3- to 4-h postfertilization (hpf). Embryos were then transferred to Pentair Aquatic Ecosystem (Apopka, FL, USA) nursery baskets (200 embryos per basket) residing in a 3-l tank in a ZebTec Recirculation Water Treatment System (Tecniplast USA, Easton, PA, USA) and raised until 72 hpf. The room housing the recirculation system was kept on a 14:10-h light: dark cycle and water temperature was maintained at 28.5 ± 0.5°C. System temperature, conductivity (900–1000 µS), and pH (7.2–7.6) were measured every day. Ammonia, nitrate, nitrite, and chlorine were measured weekly and always kept within acceptable parameters. All procedures were performed in accordance with the Canadian Council of Animal Care guidelines.

### Behavioral analysis

72 hpf larvae were individually transferred to a 48-well microtiter plate in 450 µl of a HEPES buffered E3 (HE3) medium (E3 medium, 10 mM HEPES, pH 7.2). For these experiments, subphenotypic concentrations of BPA (5 and 20 µM) and TBBPA (0.5 and 1 µM) were used. Each well then received 50 µl of a 10 X stock of test chemical or carrier control (CC), plates were sealed, and incubated at 28.5°C without media replenishment for 48 h. At 120 hpf, the film was removed from the plates, and the plates placed in a DanioVision larval tracking system with EthoVisionXT17 software (Noldus Information Technology Inc., VA, USA). Activity was recorded in 1-min bins using dynamic subtraction at 28.5° C under lighted conditions (15 µmol m^−2^ s^−1^) for 90 minutes, followed by alternating 5-min dark/light cycles. Dead or phenotypically abnormal larvae were removed from analysis and each larvae represented an independent measurement. Three replicates of 12 larvae were used in each experiment. In addition to total distance traveled (mm/min), changes in well location were calculated during the dark phases. Each well (arena) was divided into 2 zones, a centered inner circle of 6 mm (Inner), and a remaining 2 mm ring (Outer) extending from the edge of the inner zone to the well edge (inner zone = ∼36% of total area). The cumulative duration (CD) of the time spent in the inner zone was calculated and expressed as a percent of total time. Statistical analysis was performed using 1-way ANOVA with a Dunnett’s multiple comparisons test.

### Transcriptomics sampling

Transcriptomic sampling and sequencing were performed as described previously (Morash et al. 2023). Briefly, 72 hpf larvae were individually transferred to a 96-well round well microtiter plate in 270 µl of HE3 medium. Each well then received 30 µl of a 10 X stock of test chemical at their previously calculated (Achenbach et al. 2020) EC_20_ value (BPA 48.3 µM and TBBPA 2.92 µM) with a 10-fold dilution series. 0.5% DMSO was used as a CC. Plates were sealed and incubated at 28.5°C without media replenishment. 20 phenotypically normal larvae were collected at 120 hpf (48 h of exposure) into Corning Netwell baskets and rinsed 5 times in CC media, followed by once in Milli-Q type I water and collected into microcentrifuge tubes. After removing all residual water, larvae were flash-frozen on dry ice and stored at −80°C. This procedure was repeated 3 separate times to generate the 3 distinct replicates.

### RNA isolation and sequencing

Total RNA was isolated using the Norgen BioTek Corp Total RNA Purification kit (Norgen Cat No. 17200, Thorold, ON, Canada) with the optional RNase-Free DNase I kit (Norgen Cat No. 25710, Thorold, ON, Canada). Total RNA was quantitated by spectroscopy with average recoveries of BPA GBT—174.68 µg/ml (71.16–326.12), and TBBPA GBT—258.65 µg/ml (181.5–358.48). RNA integrity was determined on an Agilent Bioanalyzer using an Agilent RNA 6000 pico kit, RIN values averaged 8.7. 1 µg of total RNA was used to prepare sequencing libraries using the Illumina Truseq Stranded mRNA kit and sequenced on an Illumina Hiseq 2500 using High Output V4 2×125 bp chemistry. Library sizes averaged ∼12 M reads per sample. Reads were aligned to the zebrafish genome (GRCz11) using STAR under default parameters (Dobin et al. 2013). Approximately 84% of all reads were uniquely mapped to the genome, whereas 12.6% of reads mapped to multiple loci and ∼2.5% of reads were not mapped. Sequencing data was uploaded to the NCBI Gene Expression Omnibus repository (GSE240125). Key alignment statistics are shown in Table S1.

### Differential gene expression analysis

Raw reads were trimmed using cutadapt (Martin 2011), quality control assessed using Fastqc (Andrews 2010), and reads per gene calculated using STAR aligner (Dobin et al. 2013; Dobin and Gingeras 2015) in quant mode. Read counts from STAR were imported into DESeq2 (Love et al. 2014) for all differential gene expression (DEG) analyses using an adjusted *P*-value false discovery rate (FDR) of 0.05 (Benjamini and Hochberg) and a log-fold change of 1.5. The design parameter for DESeq2 was set as “∼replicate+condition” to normalize within samples. Results of pairwise comparisons for BPA and TBBPA are shown in Files S1 and S2, respectively. Volcano plots were generated in R (R Core Team (2022). R: A language and environment for statistical computing. R Foundation for Statistical Computing, Vienna, Austria), heatmaps using pHeatmap (Kolde 2012) (pheatmap: Pretty Heatmaps. R package version 1.0.12). Distance measure was performed using the Pearson correlation, with a normalized count threshold of 25, and graphed as log2 (EC_20_/CC) with no row scaling.

### Gene ontology analysis

DEGs identified above were used for gene ontology (GO) term analysis using g:Profiler (https://biit.cs.ut.ee/gprofiler/gost) with default settings except using “All Genes” and a Benjamini–Hochberg FDR of 0.05. Selected GO-terms, Reactome, and the KEGG pathways reported herein were manually curated based on their statistical significance (most significant) and their relation to previously described phenotypic effects. For the sake of clarity, terms and pathways with term sizes >1000 were not reported in the results. Full lists are included in Files S3 and S4.

### BMD curve fitting and POD calculations

Normalized quantile data from DESeq2 was imported into BMDExpress 2.3 (Phillips et al. 2019). Imported data was prefiltered using the Williams Trend Test (1.5-fold cut-off) and the resulting genes subjected to BMD curve fitting using all fitting models. Postfiltering was performed to remove curves with a ratio of upper and lower confidence intervals >40 (BMDU/BMDL>40), a BMD greater than the highest concentration tested, and a BMD <10-fold below the lowest tested concentration.

The 10th percentile of the postfiltered BMD values was calculated and expressed as the transcriptomic POD (tPOD) (10th). The same postfiltered BMD values were also analyzed for modes and antimodes using a custom R script (Page-Lariviere et al. 2019). The first mode is expressed as the tPOD (mode). Mode graphs are shown in Fig. S1. The genes comprising the first mode grouping were subjected to GO-term analysis as described above, the results are shown in Files S5 and S6.

## Results

### Behavioral analysis

**Fig. 1.**
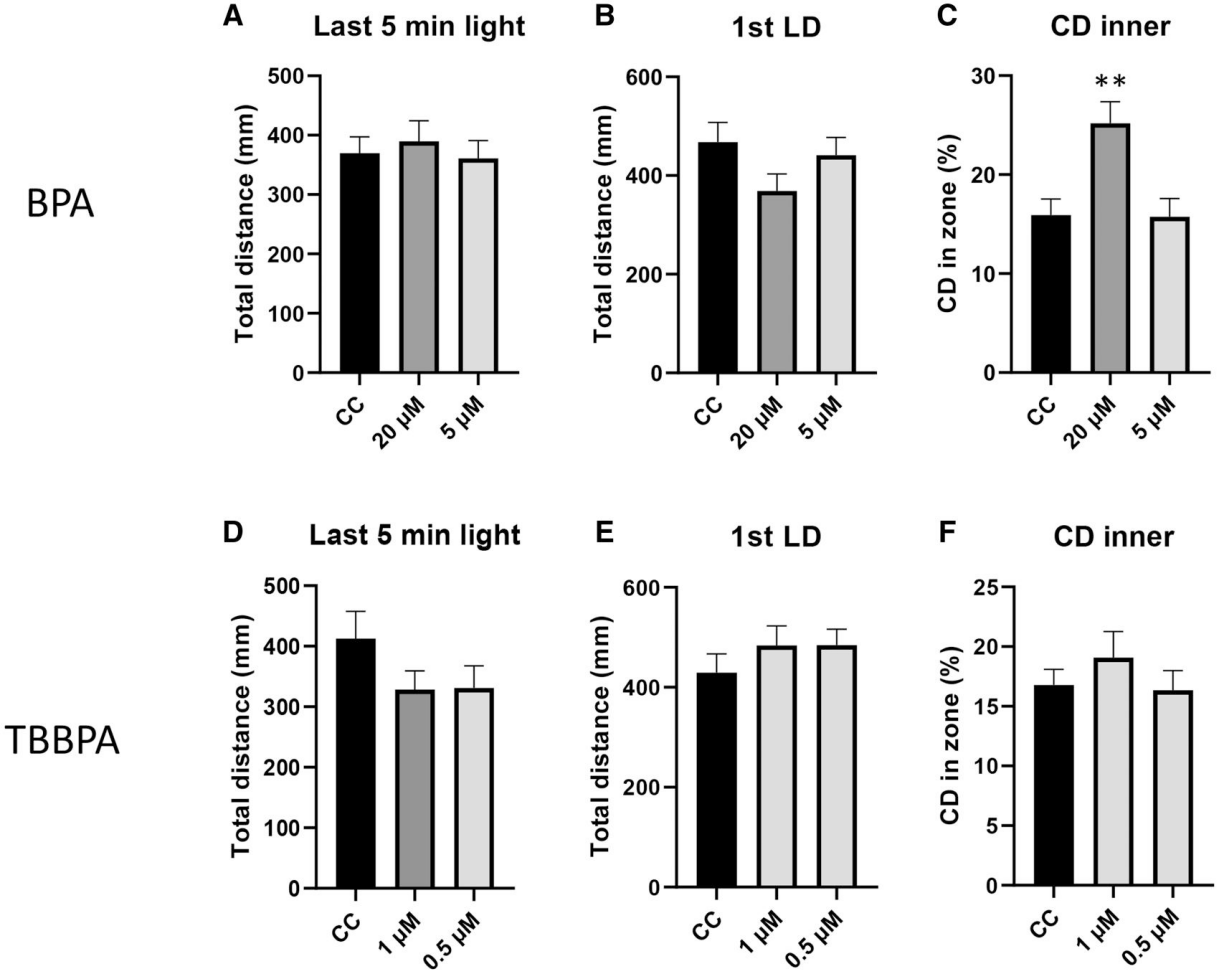
Behavioral analysis of BPA (a–c) and TBBPA (d–f) treated larvae. Total distance traveled over the last 5 min of the acclimatory baseline phase (last 5 min light) (a and d), and the total distance traveled over the first 5 min dark phase—the previous 5-min light phase (first LD) (b and e). Also, the cumulative duration (CD) of time spent in the center of the arena during the 5-min dark phase was assessed (c and f). All concentrations are in micromolar. **P < 0.01. *n* = 3, 12 larvae per replicate.

Because of conflicting reports in the literature, we initially assessed whether BPA and TBBPA were able to induce behavioral alterations. When compared with their vehicle control, there was no significant change in the baseline distance traveled or in the initial startle response for either BPA (Fig. 1A and B) or TBBPA (Fig. 1D and E). However, BPA induced a change in the thigmotactic response (see discussion), which was measured as an increased time spent in the center zone of the well during the first dark phase (Fig. 1C). During the dark phase, vehicle control larvae spent approximately 15.9 ± 1.6% of the time in the inner zone, compared with 25.2 ± 2.2% (*P* < 0.01) in the inner zone for BPA-treated larvae. TBBPA exposure did affect the thigmotactic response (Fig. 1F).

#### Differential gene expression

**Fig. 2.**
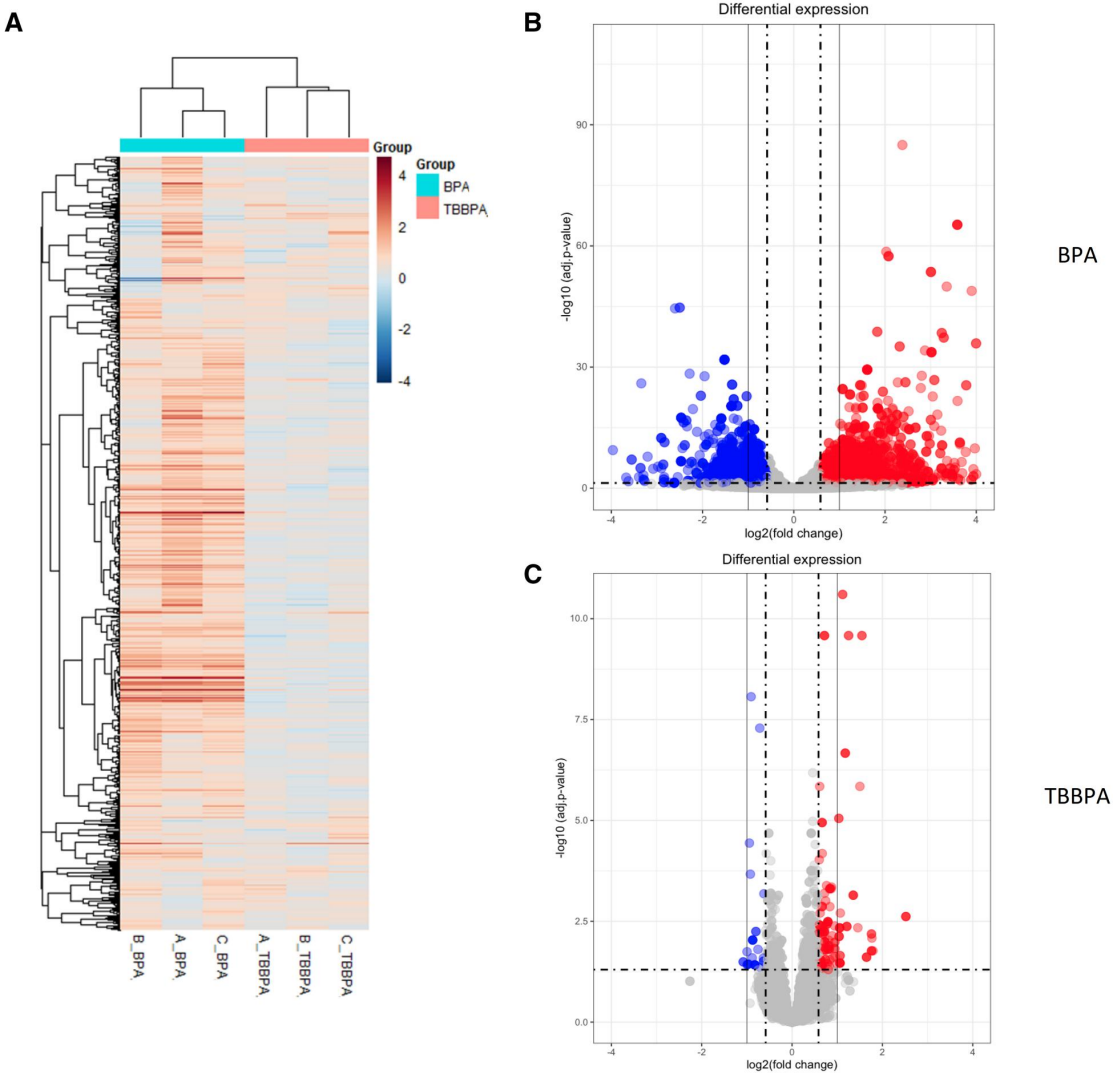
Distribution of Differentially Expressed Genes (DEGs) in BPA and TBBPA identified in the GBT assay. a) Heatmap of triplicate (A, B, and C) BPA and TBBPA samples. b and c) Graphs demonstrate the DEGs with a q < 0.05 and a positive (red) or negative (blue) fold change of 1.5 (hashed vertical line) or 2 (light gray line). Light gray points did not meet the cut off criteria. n = 3, 20 larvae per sample.

DEG was determined by pairwise comparison of the EC_20_ values (previously calculated, see Materials and methods) and CC samples using DESeq2. Statistically significant DEGs were identified using a fold-change of 1.5 (log2 = 0.585), and an adjusted *P*-value (q)<0.05. BPA exposure produced 2824 DEGs, whereas TBBPA induced markedly fewer (91) (Fig. 2). For the TBBPA testing, ∼50% (45 of 91) of the DEGs identified were also present in the BPA samples. Increasing the stringency of the fold-change cutoff to 2 resulted in a sharp decrease in DEGs: BPA (1457) and TBBPA (21) and so a 1.5-fold cutoff was used for downstream applications.

#### Gene ontology

As expected, the ∼2800 DEGs induced by BPA exposure resulted in a considerable number of GO-terms (File S3), many corresponding with previously demonstrated phenotypic outcomes of BPA exposure, including lipid absorption, cardiac function, and ROS (see Table 1). Additionally, a significant number of terms related to GABAnergic neurons, synapse formation, and neuron function were identified. Analysis of the DEGs revealed changes to a number of thyroid-related genes: thyrotropin-releasing hormone (*trh*), parathyroid hormone 1 b (*pth1b*), parathyroid hormone 2 (*pth2*), parathyroid hormone 2 receptor a (*pth2ra*), thyroid hormone receptor alpha a (*thraa*), iodothyronine deiodinase 3 b (*dio3b*), and iodothyronine deiodinase 1 (*dio1*). Similarly, genes involved in estrogenic signaling: estrogen-related receptor gamma a (*esrrga*), estrogen receptor binding site associated antigen 9 (*ebag9*), aromatase (*cyp19a1b*), were affected, and the Reactome term R-DRE-9009391 (Extra-nuclear estrogen signaling) was populated by 14 of the DEGs.

**Table 1.** Select GO, KEGG, and Reactome (REAC) terms related to previously described phenotypic effects ascribed to BPA and TBBPA exposure.

BPA				
	Source	Term name	Term ID	Adj *P*-value
**Lipid**	GO: MF	Lipid binding	GO:0008289	0.0001
	GO: MF	Lipoprotein particle binding	GO:0071813	0.0046
	KEGG	Fatty acid metabolism	KEGG:01212	0.0082
	REAC	Metabolism of lipids	R-DRE-556833	0.0119
	GO: BP	Lipid transport	GO:0006869	0.0328
**Cardiac**	KEGG	Adrenergic signaling in cardiomyocytes	KEGG:04261	1.5E-09
	KEGG	Cardiac muscle contraction	KEGG:04260	3.1E-07
	GO: BP	Vasculature development	GO:0001944	0.0387
	GO: BP	Heart development	GO:0007507	0.0441
	GO: BP	Regulation of blood pressure	GO:0008217	0.0441
**ROS**	GO: MF	Oxidoreductase activity, acting on peroxide as acceptor	GO:0016684	9.4E-05
	GO: MF	Antioxidant activity	GO:0016209	0.0001
	GO: BP	Response to oxidative stress	GO:0006979	0.0003
	GO: BP	Cellular oxidant detoxification	GO:0098869	0.0005
	REAC	Detoxification of reactive oxygen species	R-DRE-3299685	0.0022
**GABA**	REAC	GABA synthesis, release, reuptake, and degradation	R-DRE-888590	6.4E-06
	GO: MF	GABA receptor activity	GO:0016917	3.5E-05
	GO: BP	Neuron development	GO:0048666	8.1E-05
	GO: BP	Regulation of neurotransmitter levels	GO:0001505	0.0006
	GO: MF	Postsynaptic neurotransmitter receptor activity	GO:0098960	0.0016

TBBPA				
	Source	Term name	Term ID	Adj *P*-value
**O_2_/Hb**	GO: CC	Haptoglobin–hemoglobin complex	GO:0031838	4.2E-06
	GO: MF	Haptoglobin binding	GO:0031720	7.2E-06
	GO: MF	Oxygen carrier activity	GO:0005344	7.2E-06
	GO: CC	Hemoglobin complex	GO:0005833	1.2E-05
	GO: MF	Oxygen binding	GO:0019825	5.1E-05
	GO: BP	Oxygen transport	GO:0015671	0.0001
	GO: MF	Heme binding	GO:0020037	0.0032
	GO: MF	Tetrapyrrole binding	GO:0046906	0.0033
**Detox**	GO: BP	Reactive oxygen species metabolic process	GO:0072593	0.0007
	GO: BP	Cellular oxidant detoxification	GO:0098869	0.0014
	GO: BP	Cellular response to toxic substance	GO:0097237	0.0018
	GO: BP	Cellular detoxification	GO:1990748	0.0018
	GO: BP	Detoxification	GO:0098754	0.0027
	GO: BP	Response to toxic substance	GO:0009636	0.0036
**ROS**	GO: BP	Hydrogen peroxide catabolic process	GO:0042744	6.6E-05
	GO: BP	Hydrogen peroxide metabolic process	GO:0042743	6.6E-05
	GO: MF	Oxidoreductase activity, acting on peroxide as acceptor	GO:0016684	0.0004
	GO: MF	Antioxidant activity	GO:0016209	0.0007

As noted, TBBPA exposure produced markedly fewer DEGs than BPA exposure, and thus also produced considerably fewer GO-terms (Table S4). Generally, 2 broad categories of GO-terms were represented; oxygen/hemoglobin (Hb) binding and detoxification (Table 1). Many of these terms were also identified in the BPA-treated larvae, suggesting a common mechanism. Specifically, 32 of the 48 GO/KEGG pathways identified in the TBBPA-treated group were present in the BPA group, including all of those listed for TBBPA in Table 1. In contrast to the BPA results, none of the GABA/neuron GO-terms were identified in the TBBPA-treated larvae, and no thyroid- or estrogen-related DEGs were identified.

#### BMD and tPOD calculations

In order to calculate tPODs, we initially performed a BMD analysis. BMD analysis of BPA gene expression data identified 3784 DEGs, compared with 2824 identified by DESeq2. 87.1% of the DESeq2 DEGs were also identified by BMDExpress. The lowest BMD values were used to calculate 2 tPODs based on previously published methods (Morash et al. 2023). Both calculations yielded similar values (POD (10th)—0.612 µM vs POD (mode)—0.324 µM). These values are both ∼100-fold lower than the EC_20_ value (48.3 µM).

In order to identify the most sensitive pathways to BPA exposure, we analyzed the genes nearest to the first mode of DEGs (see Fig. S1). 372 DEGs from −0.2 to −0.8 were subjected to GO-analysis. Of particular interest in this first mode group of genes is the presence of a number of thyroid hormone-related DEGs, including, thyroid hormone receptor alpha a (*thraa*), iodothyronine deiodinase 1 (*dio1*), and parathyroid hormone 1 b (*pth1b*) suggesting the thyroid system may be especially sensitive to BPA exposure. The predominant GO-terms identified in this first mode consisted of amino acid transporters, and synaptic neuron formation. The abundance of synaptic functions indicates that neurodevelopment is among the most sensitive pathways (Table 2).

**Table 2.** GO-terms identified as most sensitive after BPA exposure.

	Source	Term name	Term ID	Adj *P*-value
**Transporter**	GO: MF	Transmembrane transporter activity	GO:0022857	0.0014
	GO: MF	Glutamate: sodium symporter activity	GO:0015501	0.0057
	GO: MF	L-glutamate transmembrane transporter activity	GO:0005313	0.0216
	GO: MF	Acidic amino acid transmembrane transporter activity	GO:0015172	0.0252
	GO: MF	Amino acid: sodium symporter activity	GO:0005283	0.0287
	GO: MF	Amino acid: monoatomic cation symporter activity	GO:0005416	0.0308
	GO: BP	L-glutamate transmembrane transport	GO:0015813	0.0455
	GO: BP	L-glutamate import	GO:0051938	0.0455
	GO: BP	Acidic amino acid transport	GO:0015800	0.0455
**Synapse**	GO: BP	Trans-synaptic signaling	GO:0099537	0.0022
	GO: CC	Synaptic membrane	GO:0097060	0.0017
	GO: BP	Anterograde trans-synaptic signaling	GO:0098916	0.0022
	GO: BP	Chemical synaptic transmission	GO:0007268	0.0022
	GO: BP	Synaptic signaling	GO:0099536	0.0026
	GO: CC	Neurotransmitter receptor complex	GO:0098878	0.0084
	GO: BP	Neurotransmitter transport	GO:0006836	0.0086
	GO: BP	Regulation of synaptic plasticity	GO:0048167	0.0212
	GO: CC	Cytoskeleton of presynaptic active zone	GO:0048788	0.0271
	GO: CC	Neuron to neuron synapse	GO:0098984	0.0278
	GO: CC	Synaptic vesicle membrane	GO:0030672	0.0283
	REAC	Transmission across Chemical Synapses	R-DRE-112315	0.0285
	GO: BP	Regulation of trans-synaptic signaling	GO:0099177	0.0291
	GO: BP	Regulation of neurotransmitter levels	GO:0001505	0.0291
	GO: BP	Modulation of chemical synaptic transmission	GO:0050804	0.0291
	GO: CC	Presynaptic active zone cytoplasmic component	GO:0098831	0.0338
	GO: BP	Neurotransmitter secretion	GO:0007269	0.0455

BMD and tPOD calculations were similarly performed for TBBPA. Although DESeq2 identified only 91 DEGs, BMDExpress identified 720, and 92.3% of the DESeq2 DEGs were observed in BMDExpress. As with BPA, both tPOD calculations yielded similar values (POD (10th)—0.019 µM, POD (mode)—0.017 µM) which were ∼100-fold lower than the EC_20_ value (2.92 µM).

The 102 DEGs populating the first mode cluster (−2.2 to −1.4, see Fig. S1) were then used in GO analysis. None of these DEGs were related to thyroid or estrogen function. No GO-terms were identified, only a limited number of blood group-related Reactome terms (Plasma lipoprotein assembly, remodeling, and clearance [R-DRE-174824], ABO blood group biosynthesis [R-DRE-9033807], and Blood group systems biosynthesis [R-DRE-9033658]) were identified.

In conclusion, the tPODs calculated for both compounds were well below the EC_20_ and behavioral effect values (Table 3). In addition, the tPOD approach identified a number of the pathways that are most sensitive to these bisphenols.

**Table 3.** Summary of results.

	BPA	TBBPA
EC_20_ *	48.3 µM	2.92 µM
Behavior	20 µM	N/A
POD 10th	0.612 µM	0.019 µM
POD mode	0.324 µM	0.017 µM

* From Achenbach et al. (2020).

## Discussion

The goal of this study was to continue the development of the larval zebrafish platform as a robust tool for generating hazard data as an alternative to the rodent model for use in human health risk assessment. The transcriptomics platform potentially provides a more sensitive method to identify not only adverse effects resulting from chemical exposure but also, through direct analysis of DEGs and extrapolation from GO-term-based pathway enrichment, to strengthen interspecies ability to predict human health toxicity with greater confidence. The results of this study reveal that this transcriptomic model can be used to differentiate toxicological mechanisms between 2 similar chemicals, BPA and TBBPA. This is important for the purpose of chemical risk assessment as it helps to qualify the value of read across between similar chemicals in attempts to fill data gaps. Through transcriptomic analysis, we identified pathways involved in many of the previous phenotypic profiles described for these chemicals, and some previously unreported pathways. Importantly, we were able to identify both shared and unique pathways, attesting to the caution that is required when extrapolating between compounds with similar chemical structures. The identification of endpoints arising by endocrine-mediated MOAs is a high-priority initiative in the international regulatory community. The use of transcriptomics analysis for the evaluation of nonapical endpoints is a promising approach for the evaluation of endocrine-mediated responses (Ankley et al. 2009; Lupu et al. 2020). With respect to endocrine disruption, given that the endogenous expression of aromatase and thyroid hormones begins at 48–72 hpf (Cohen et al. 2022), evaluation of the role of BPA and TBBPA as endocrine disruptors in the later onset GBT paradigm is beneficial. In addition to selecting an appropriate exposure time frame, the chemical concentration of our exposure is also noteworthy. By selecting nonaffected larvae at or below the EC_20_, we can limit transcriptomic changes associated with gross morphological damage that may include necrosis and as such may be less related to chemical-specific toxicity. Importantly, it also reflects a standardized point at which all compounds can be tested, as opposed to an arbitrary concentration. Additionally, because toxicokinetics plays an important role in toxicity testing (Achenbach et al. 2022), by basing exposure paradigms on toxicity (EC_20_), and not on fixed concentrations, we can assure we are in a phenotypically relevant range. Our method also utilizes 2 transcriptomic analysis approaches, a comparison of the maximal subphenotypic dose response (DESeq2), as well as the identification of the most sensitive pathways and the subsequent establishment of a POD (BMD). The BMD approach typically yields a higher number of DEGs (compared with pairwise comparison) because it is based on multiple concentrations, and allows for the inclusion of multimodal expression patterns, specifically at lower concentrations.

Our transcriptomic data strongly correlate with previously described zebrafish phenotypic effects attributed to BPA, and confirm the limited previously reported transcriptomic data (Martinez et al. 2018). Specifically, our data identified a number of cardiac and vasculature-related GO-terms induced by BPA exposure. This supports previously published BPA-mediated effects on heart development (Achenbach et al. 2020; Heredia-García et al. 2022), vascular toxicity, and BPA-induced vascular development (Ji et al. 2022) in zebrafish. Similarly, previously reported issues regarding yolk and lipid defects including lipid uptake (Mu et al. 2022), metabolism (Sun et al. 2020), and lipid production (Ortiz-Villanueva et al. 2018) were also well-represented in the transcriptomic data. BPA-induced reactive oxygen/nitrogen species production is well-documented (Biswas et al. 2020; Haridevamuthu et al. 2022; Qian et al. 2023) and our data revealed a number of reactive oxygen and nitrogen detoxification pathways, antioxidant, and hydrogen peroxide pathways which support these findings.

Of particular interest was the identification of a number of GABA and neuronal synapse-specific GO-terms, and that the BMD analysis revealed these to be among the most sensitive pathways to BPA exposure. Larval exposure to BPA has been shown to cause transcriptional changes in GABA levels in later juveniles along with behavioral changes (Naderi et al. 2022). Additionally, BPA affects Cypin function, which is a synaptic vesicle protein and has a minor behavioral effect (Liu et al. 2023). At a more structural level, BPA causes brain morphology deficits (Yang et al. 2023), neuronal development effects (Gu et al. 2022), along with increases in the expression of a number of Developmental Neurotoxicity (DNT)-related genes (Gyimah et al. 2021). Depending on the exposure paradigm, there are conflicting reports regarding the BPA-induced behavioral changes with reports of hyperactivity (Bai et al. 2023), hypoactivity (Gu et al. 2021), reductions in high-velocity motion (Wang et al. 2021) and social behaviors (Scaramella et al. 2022). Our transcriptomic data revealed a number of sensitive synaptic and GABAnergic DEGs which appear to translate to behavioral alterations, specifically decreased thigmotactic behavior. Upon transition from light to dark in their environment, zebrafish larvae typically increase their activity and remain in the outer edges of the test arena (Schnorr et al. 2012). This thigmotactic behavior is an anxiety response which is attenuated by GABA agonists like diazepam (Schnorr et al. 2012). It is therefore tempting to speculate the changes in GABA-related gene expression are responsible for the antianxiolytic behavior observed upon BPA exposure. A number of the previously described behavioral effects attributed to BPA exposure have been linked to ROS (Sahoo et al. 2020; Yang et al. 2023), which could also be supported by our transcriptomic data.

Despite the chemical similarities, TBBPA had a significantly different transcriptomic response when compared with BPA. At the EC_20_, TBBPA displayed only ∼5% of the DEGs as BPA. Of note, 2/3 of the GO-terms identified for TBBPA were also present in BPA-treated samples (32/48). Interestingly the predominant pathways affected (oxygen binding, detoxification, and ROS) are nonobservable, and therefore without transcriptomics would only have been observed through molecular methods. Many of the oxygen binding and detoxification pathways were also present in the BPA-treated larvae, indicating shared mechanisms of toxicity. Interestingly both BPA and TBBPA have been shown to be metabolized via the same detoxification pathways (Achenbach et al. 2022). There is limited previously published phenotypic information regarding TBBPA exposure. TBBPA has been shown via transcriptomics to affect early organ morphogenesis at 1.5 µM at 52 hpf (Pang et al. 2022), as well as edema (Usenko et al. 2016) and vascular malformations (Li et al. 2016). Additionally, TBBPA displays hematopoietic toxicity in early embryos, with upregulation of epidermal and blood genes (Pang et al. 2020), which aligns with our data. Blood group-related GO-terms made up the most sensitive pathways, demonstrating the hematopoietic effects of TBBPA. Although the behavioral role of TBBPA in zebrafish is contested (for a review, see Kacew and Hayes 2020), some groups have reported behavioral effects (Chen et al. 2021a; Noyes et al. 2015). Conversely to the effects seen after BPA exposure, we did not observe any antithigmotactic behavior caused by TBBPA in the GBT model. Although neither chemical caused changes in total distance or startle response, these behavioral experiments are not an exhaustive evaluation of all locomotory functions, and further studies may reveal chemical-specific effects.

Our transcriptomic approach also clearly confirms the previously reported estrogenic and goitrogenic endocrine-disrupting potential of BPA. We identified expression changes in estrogen-related receptors and aromatase at the EC_20_ value. This is in agreement with several reports of BPA inducing production of 17β-estradiol (Karim et al. 2022) and in the expression of endocrine-related genes including ERα, aromatase, and *vtg* (Chen et al. 2021b), and increased yolk sac area, hatching defects, and a number of cranial cartilage defects via the estrogen receptor (Huang et al. 2021). Conversely, we did not detect estrogenic activity in TBBPA-treated larvae, a finding which has been previously reported (Song et al. 2014), and BPA activates Cyp19a1b through the estrogen receptor, but TBBPA does not (Kubota et al. 2023). The goitrogenic activity of BPA was also clear from transcriptomic results, and importantly, many of these genes were among the most sensitive as shown by BMD modeling. We did not observe significant changes in thyroid-related genes upon TBBPA exposure, although this does not rule out the possibility that TBBPA signaling directly through the thyroid hormone receptors is occurring. In fact, considerable evidence exists in the literature that this may be the case (Chan and Chan 2012; Baumann et al. 2016; Zhu et al. 2018). It should be noted we were able to detect several thyroid-related transcripts, but none were significantly differentially expressed using our exposure paradigm.

## Conclusion

In conclusion, we compare and contrast subphenotypic markers of toxicity of 2 chemicals with the same structural backbone; BPA and TBBPA, the results of which provide strong rationale for a cautionary approach in the use of read across to fill data gaps in chemical risk assessment. As a regulatory tool for quantitative chemical risk assessment, this *in vitro* larval zebrafish transcriptomics approach represents what may become an integral component of using embryo/larval zebrafish as a NAM within an integrated approach for testing and assessment in a manner similar to a traditional “weight of evidence” collection of all relevant data and information to determine risk to human health and the environment following chemical exposure.

## Supplementary Material

kfae063_Supplementary_Data
