# Supplementary material for: Comparative toxicological assessment of 2 bisphenols using a systems approach: evaluation of the behavioral and transcriptomic responses of Danio rerio to bisphenol A and tetrabromobisphenol A
Source: Toxicol Sci. 2024 May 10;200(2):394–403. doi: 10.1093/toxsci/kfae063 (PMC11285168; doi:10.1093/toxsci/kfae063)
Supplement: kfae063_Supplementary_Data [file kfae063_supplementary_data.zip › kfae063_Supplementary_Data/toxsci-24-0045-File004.docx]

Supplementary Data.

Supplemental Table 1. Selected parameters of STAR alignment and abundance quantification. See below.

Supplemental Figure 1. Mode calculations for Bisphenol A (BPA) and Tetrabromobisphenol A (TBBPA). See below.

Supplemental file 1 – Bisphenol A EC_20_ vs CC_20_ DEGs using a 1.5-fold change cutoff and a padj (q) < 0.05.

Supplemental file 2 – Tetrabromobisphenol A EC_20_ vs CC_20_ DEGs using a 1.5-fold change cutoff and a padj (q) < 0.05.

Supplemental file 3 – gProfiler result files for Bisphenol A using DEGs with Fold Change > 1.5 and q < 0.05.

Supplemental file 4 – gProfiler result files for Tetrabromobisphenol A using DEGs with Fold Change > 1.5 and q < 0.05.

|  | BPA | TBBPA |
| --- | --- | --- |
|  | Avg | Avg |
| Number of input reads | 11363581 | 13425112 |
| UNIQUE READS: |  |  |
| Uniquely mapped reads number | 9580935 | 11313884 |
| Uniquely mapped reads % | 84.26 | 84.28 |
| Average mapped length | 123.72 | 123.95 |
| Number of splices: Total | 4113259 | 4787105 |
| Number of splices: Annotated (sjdb) | 4025708 | 4698165 |
| Mismatch rate per base, % | 0.39 | 0.45 |
| Deletion rate per base | 0.0004 | 0.0005 |
| Deletion average length | 2.6 | 2.5 |
| Insertion rate per base | 0.0002 | 0.0003 |
| Insertion average length | 2.28 | 2.11 |
| MULTI-MAPPING READS: |  |  |
| Number of reads mapped to multiple loci | 1437468 | 1700564 |
| % of reads mapped to multiple loci | 12.7 | 12.67 |
| UNMAPPED READS: |  |  |
| % of reads unmapped: too many mismatches | 0 | 0 |
| % of reads unmapped: too short | 2.56 | 2.56 |
| % of reads unmapped: other | 0.24 | 0.27 |
| CHIMERIC READS: | 0 | 0 |

Supplemental Table 1. Selected parameters of STAR alignment and abundance quantification.


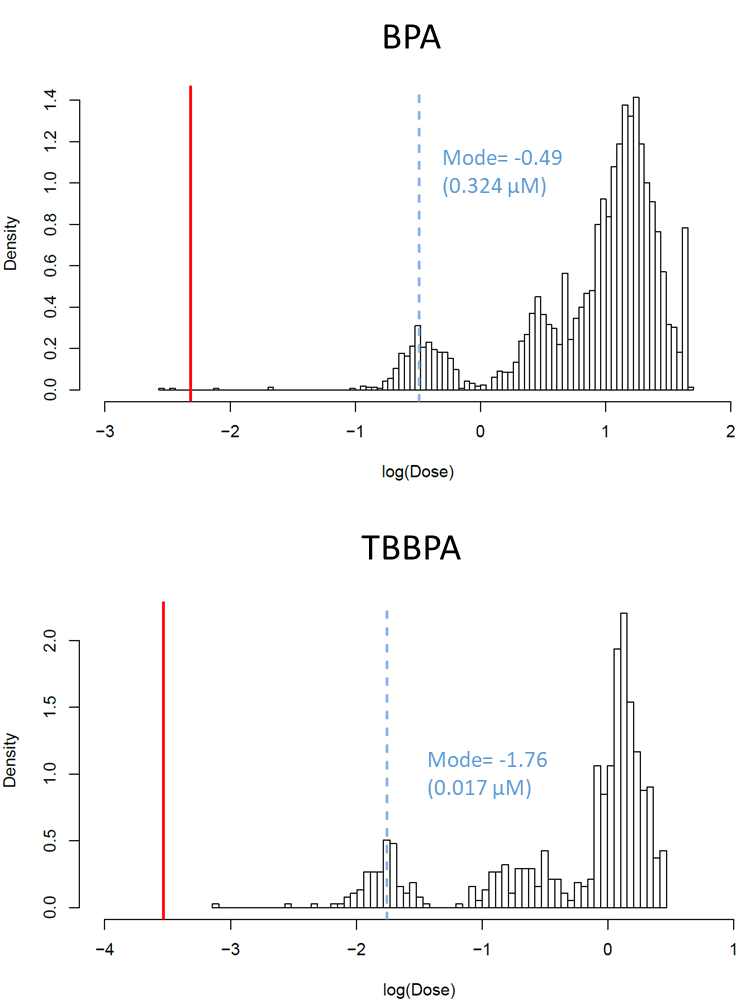


Supplemental Figure 1. Histograms of mode values obtained from BMDExpress 2.3. Light blue hashed line indicates the first calculated mode, as well as the corresponding concentration.
